# Blood Concentrations of Designer Benzodiazepines: Relation to Impairment and Findings in Forensic Cases

**DOI:** 10.1093/jat/bkaa043

**Published:** 2020-05-05

**Authors:** Gunhild Heide, Gudrun Høiseth, Gerrit Middelkoop, Åse Marit Leere Øiestad

**Affiliations:** Department of Forensic Sciences, Oslo University Hospital, 0424 Oslo, Norway; Department of Forensic Sciences, Oslo University Hospital, 0424 Oslo, Norway; Center for Psychopharmacology, Diakonhjemmet Hospital, 0319 Oslo, Norway; Department of Forensic Sciences, Oslo University Hospital, 0424 Oslo, Norway; Department of Forensic Sciences, Oslo University Hospital, 0424 Oslo, Norway

## Abstract

The use of designer benzodiazepines appears to be increasing in many countries, but data concerning blood concentrations are scarce, making interpretation of concentrations difficult. The aim of this study was to report blood concentrations of clonazolam, diclazepam, etizolam, flualprazolam, flubromazepam, flubromazolam and phenazepam and to investigate the relationship between blood concentrations and impairment. The concentration data are from blood samples collected from living cases (apprehended drivers and other drug offences) and medico-legal autopsies. The blood samples were analysed for the seven designer benzodiazepines mentioned above by ultra high performance liquid chromatography–tandem mass spectrometry. Positive cases from between 1 June 2016 and 30 September 2019 were included. Blood concentrations and the conclusion from a clinical test of impairment (when available) are reported. The presented seven benzodiazepines were detected in a total of 575 cases, where 554 of these cases concerned apprehended drivers or other criminal offenders. The number of findings and the median (range) concentrations were as follows: clonazolam, *n* = 22, 0.0041 mg/L (0.0017–0.053 mg/L); diclazepam, *n* = 334, 0.0096 mg/L (0.0016–0.25 mg/L); etizolam, *n* = 40, 0.054 mg/L (0.015–0.30 mg/L); flualprazolam, *n* = 10, 0.0080 mg/L (0.0033–0.056 mg/L); flubromazepam, *n* = 5, 0.037 mg/L (0.0070–0.70 mg/L); flubromazolam, *n* = 20, 0.0056 mg/L (0.0004–0.036 mg/L); and phenazepam, *n* = 138, 0.022 mg/L (0.0018–0.85 mg/L). A designer benzodiazepine was the only drug detected with relevance for impairment in 25 of the 554 living cases. The physician concluded with impairment in 19 of the 25 cases. Most of the concentrations in these cases were relatively similar to or higher than the median reported concentrations. The most frequent other drugs detected were amphetamine, tetrahydrocannabinol, clonazepam and methamphetamine. The presented blood concentrations can be helpful with the interpretation of cases involving one or more of these seven benzodiazepines. The results indicate that concentrations commonly observed in forensic cases are associated with impairment.

## Introduction

Over the past years, there has been an increase in new psychoactive substances on the recreational drug market driven by globalization and new technologies. The Internet makes these substances accessible independently of geography and social network. Designer benzodiazepines are one group of new psychoactive substances with an increasing use in many countries (1–5). Many of these drugs have pharmaceutical origin, even though many never have been marketed as pharmaceuticals. Some can be prescribed in certain countries; others are classified as research chemicals. Designer benzodiazepines can also be used to make fake tablets of common benzodiazepine medicines, which are sold on the illicit market (2, 3, 6).

**Figure 1:**
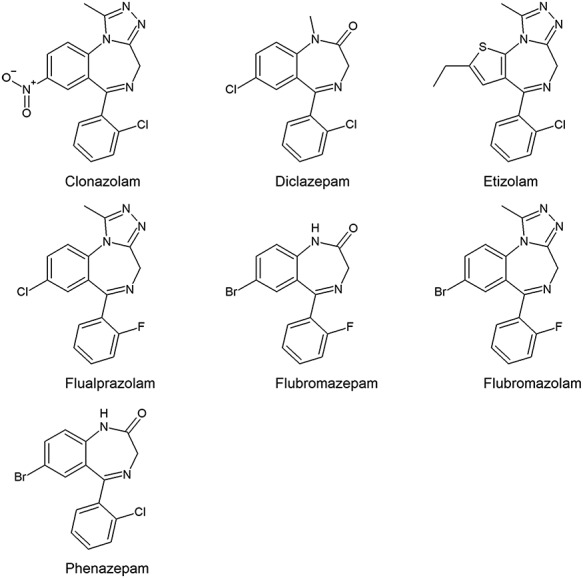
The chemical structures of the seven designer benzodiazepines.

Phenazepam and etizolam are available as medicines in some countries and are therefore not designer benzodiazepines in a strict sense but are considered as the starting point of the phenomenon (7). For simplicity, etizolam and phenazepam are also included in the term designer benzodiazepines in this article. Phenazepam and etizolam have been sold as legal highs on the drug market but are now scheduled in many countries. The medical use of phenazepam and the benzodiazepine-analogue etizolam is limited to a few countries; phenazepam is a prescription medication in Russia and some Baltic states, while etizolam is prescribed in countries such as Japan, Italy and India. Both drugs are abused at a considerable scale. Pyrazolam started to show up on the Internet as a research chemical in 2012 and was later followed by diclazepam and flubromazepam. More designer benzodiazepines, such as, clonazolam, flubromazolam and flualprazolam, have appeared over the past few years (2–4, 8, 9). According to a report published in 2018, a total of 14 new benzodiazepines have been reported to the EU Early Warning System since 2015, and EMCDDA are currently monitoring 23 new benzodiazepines (10). The chemical structures of the designer benzodiazepines included in this study are shown in Figure 1.

It would be expected that designer benzodiazepines have many of the same effects as regular benzodiazepines, which includes anxiolytic effects, hypnotic effects, sedation, muscle relaxation and anticonvulsive effects (7). However, many designer benzodiazepines are more potent compared to several of the more traditional benzodiazepines, and this may increase the risk of hazardous use. This is especially true when it comes to fake medicines, where the user does not know the true identity of the compound or the actual dose. There are also significant variations between the different designer benzodiazepines when it comes to effective doses and duration of action (1–3). Reports of serious or fatal intoxications exist for designer benzodiazepines, either as single intoxication or in combination with other drugs (5, 11–21).

Toxicological interpretation of concentrations of new designer benzodiazepines in blood is somewhat demanding because published data concerning human drug concentrations in biological samples are scarce, especially in relation to impairment (2, 3, 11, 14, 22–27).The aim of the present study was to report blood concentrations and their relation to impairment for clonazolam, diclazepam, etizolam, flualprazolam, flubromazepam, flubromazolam and phenazepam in forensic cases.

## Methods

### Study group

In Norway, ~8,500 blood samples are taken each year from drivers suspected of driving under the influence of drugs (DUID) or other drug offenders (mainly related to acts of violence), hereafter referred to as living cases. All the samples are analysed for ethanol and an array of common illicit or prescribed drugs at the Department of Forensic Sciences, Oslo University Hospital. In addition, ~2,000 blood samples from medico-legal autopsy cases are analysed in the same laboratory each year. Blood samples from living subjects are collected in 5 mL BD Vacutainer evacuated glass tubes (BD Diagnostics, Plymouth, UK) containing 20 mg sodium fluoride and 143 I.U. heparine. In autopsy cases, whole blood from the femoral vein are collected in 25 mL Sterilin® polystyrene tubes with polyethene screw caps (Bibby Sterilin, Staffordshire, UK) containing 0.3 mL 67% (w/v) potassium fluoride solution. The blood samples are stored in a cooler at 4°C for up to 2 weeks, until all analyses are complete. The present material represents cases involving clonazolam, diclazepam, etizolam, flubromazepam, flubromazolam and phenazepam submitted for analyses in the period 1 June 2016 to 30 September 2019. Flualprazolam was added to the analysis repertoire in December 2018, and flualprazolam positive cases after this date are also included in this study.

In the present study, mono cases were defined as cases where one of the designer benzodiazepines was the only drug detected or where other drugs were not found to be contributing to impairment. For a selection of the most common illicit and medicinal drugs in Norway, there exist *per se* limits that correspond to blood alcohol concentrations of 0.2‰ (28). The criterion for a drug not to be regarded as contributing was mainly that the concentration was below the *per se* limits (given in Supplementary Table I). In cases where there was not a preexisting legal limit, the drug concentration was considered irrelevant for impairment if it was in a sub-therapeutic range.

### Analytical methods

Blood samples were screened for alcohol using an enzymatic method (29) and for a broad repertoire of illegal drugs and medicines, including a selection of benzodiazepines and z-hypnotics, opiates and opioids, amphetamines and other psychostimulants and tetrahydrocannabinol (THC), by a modified version of an ultra high performance liquid chromatography–tandem mass spectrometry (UHPLC–MS-MS) method (30). Etizolam, diclazepam, flubromazepam, clonazolam and flubromazolam were screened by the method the whole study period, whereas flualprazolam was included at the end of 2018. An acidic mobile phase consisting of methanol and ammonium formate buffer pH 3.1 was used for the screening method and separation was performed on an Acquity HSS T3-column (Waters, Milford, MA, USA). Detection was performed with Xevo-TQ-S MS instruments (Waters).

In the first part of the study period, confirmation analyses of designer benzodiazepines were performed using a UHPLC–MS-MS method with the same gradient as the screening method but with an alkaline mobile phase consisting of 5 mM ammonium bicarbonate buffer pH 8 and methanol (22). An Acquity UPLC BEH Phenyl-column (Waters) was used for chromatographic separation. Flualprazolam was also quantitated by this method. Diazepam-d_5_ was used as internal standard. From December 2017, confirmation analysis (except flualprazolam) was performed based on the method by Sauve et al. (31) where a mobile phase consisting of ammonium formate pH 5 and acetonitrile was used to separate the compounds on a BEH C18-column (2.1 × 100 mm, 1.7 μm, Waters). The method was transferred from Premier to Xevo TQ-S (Waters) in June 2018, with minor adjustments to the method (flow of 0.5 mL/min, temperature 60°C, injection volume 0.1 μL). Nitrazepam-d_5_ was used as internal standard for clonazolam, flubromazolam, etizolam and diclazepam and flunitrazepam-d_7_ as internal standard for flubromazepam and phenazepam. Diclazepam-d_4_ was later added as internal standard for diclazepam, and etizolam-d_3_ replaced nitrazepam-d_5_ as internal standard for clonazolam, etizolam and flubromazolam. The quantitative results are based on duplicate analyses for both methods.

Clonazolam, flubromazolam, etizolam and the internal standards diazepam-d_5_, nitrazepam-d_5_ and flunitrazepam-d_7_ were supplied by Chiron (Trondheim, Norway). Phenazepam was supplied by Chiron and Lipomed (Arlesheim, Switzerland), diclazepam by Chiron and LGC Standards (Luckenwalde, Germany) and flubromazepam by Chiron and Cerilliant Corporation (Round Rock, TX, USA). Flualprazolam was supplied by Cayman Chemical (Ann Arbor, MI, USA) and etizolam-d_3_ by Cerilliant Corporation. Stock standard solutions of the analytes were prepared in methanol, and working standard solutions were prepared in water or water/methanol (80:20) from the stock solution. Five calibration samples were prepared from whole blood spiked with working standard solutions at the appropriate concentration range. Samples were prepared either using a liquid–liquid extraction with ethyl acetate/heptane mixture (4:1 v/v), after addition of borate buffer pH 11 (32) or by liquid–liquid extraction with tert-butyl methyl ether after addition of borate buffer pH 11 (31).

The MRM transitions, cone voltages and collision energies used for the measurement of the compounds and the internal standards are provided in Table I, in addition to typical retention times. The lowest calibrators had S/N-ratio > 10 for both quantifier ion and qualifier ion for all compounds. Quality control (QC) samples were prepared independently at two concentration levels. Quadratic calibration curves, weighted x (1/x), were used. Correlation coefficients were greater than 0.998, and QC sample results were within 15% from the nominal values. The screening and the conformation results showed a good accordance (±20%).

**Table I:** MRM Transitions, Cone Voltages, Collision Energies and Retention Times for Compounds and Internal Standards (IS)

Compound	MRM transitions (*m/z*)	Cone voltage (V)	Collision energy (eV)	Retention time (min)	IS used
Clonazolam	354.0 > 308.0/326.0	40	20	2.11	Etizolam-d_3_/ nitrazepam-d_5_
Diclazepam	321.0 > 154.0/227.0	40	31/33	5.38	Diclazepam-d_4_/ nitrazepam-d_5_
Etizolam	343.0 > 314.0/206.0	40	26/24	3.99	Etizolam-d_3_/ nitrazepam-d_5_
Flualprazolam	327.2 > 299.1/292.1	45	25	3.80^a^ (2.81)^b^	Diazepam-d_5_
Flubromazepam	335.0 > 186.0/179.0	45	45/40	3.67	Flunitrazepam-d_7_
Flubromazolam	371.0 > 292.0/343.0	60	31/37	3.06	Etizolam-d_3_/ nitrazepam-d_5_
Phenazepam	351.1 > 179.1/206.1	50	40/37	4.24	Flunitrazepam-d_7_
Diazepam-d_5_	290.1 > 198.1	40	30	4.24^a^	
Diclazepam-d_4_	325.0 > 154.0	40	30	5.33	
Etizolam-d_3_	348.0 > 319.0	40	24	3.97	
Flunitrazepam-d_7_	321.1 > 275.1	35	25	3.05	
Nitrazepam-d_5_	287.0 > 185.0	50	30	2.25	

^a^ Flualprazolam and diazepam-d_5_ were analysed with a different method than the other compounds given in the table.

^b^ Flualprazolam has been added to the same method as the other compounds at a later date, and the corresponding retention time is given in parenthesis for comparison.

Cut-off values for the designer benzodiazepines were as follows: clonazolam 0.0014 mg/L, diclazepam 0.0016 mg/L, etizolam 0.014 mg/L, flualprazolam 0.0016 mg/L, flubromazepam 0.0033 mg/L, flubromazolam 0.00037 mg/L and phenazepam 0.0017 mg/L. They were chosen to be at or above the analytically determined limit of quantification (LOQ) for each compound. The exact levels were based upon information about the compounds from the literature or set to a comparable level as other benzodiazepines already analysed at our laboratory.

### Clinical test of impairment

A clinical test of impairment (CTI) is performed by a physician in most living cases in relation to drawing a blood sample, preferably shortly after apprehension. The CTI consists of 25 subtests and observations related to common signs of drug impairment, which includes assessment of alertness, appearance, cognitive function, motor coordination and vestibular function. The test has been described in detail elsewhere (33). At the end of the report, the physician concludes on the level of impairment. This is based on the test results and the person’s general condition. The conclusion is one out of five possibilities: ‘not impaired’, ‘mildly impaired’, ‘moderately impaired’, ‘considerably impaired’ or, in some cases, ‘impairment impossible to determine’.

### Ethics and statistics

The collected data in this study were handled in agreement with the current data processing agreement between the Norwegian Higher Prosecuting Authority, which is the owner of forensic materials in Norway, and the Department of Forensic Sciences, Oslo University Hospital. Only anonymous data were available to the authors.

The statistical analyses were performed using IBM SPSS® Software version 25.0. The data were not normally distributed. Median, 25th percentile, 75th percentile, IQR (interquartile range) and range values are reported for the continuous variables, and frequency distributions were used for the categorical variables. For assessing the relationship between drug concentrations and degree of impairment, Spearman’s rank correlation test was used.

## Results

During the study period of 3 years and 3 months, almost 21,500 DUID cases, 5,700 cases involving other crimes and 6,500 autopsy cases were included. Clonazolam, diclazepam, etizolam, flualprazolam, flubromazepam, flubromazolam and phenazepam were detected in a total of 575 cases during the study period. Most of these cases involved living subjects (*N* = 554). In ~81% of the living cases, the sample giver was between 20 and 44 years of age, with the most frequent age group being 30–34 years (21%). Most of them were men (87%). In 13 cases, 2 of the designer benzodiazepines were present in the same sample. Flualprazolam, flubromazolam and phenazepam were all detected in the same blood sample in one additional case. The total number of detections was 569 in the living cases. Table II presents the number of detections of each drug and the individual concentrations of each benzodiazepine found in the cases involving drugged driving and other drug offences. The most prevalent designer benzodiazepine detected was by far diclazepam, followed by phenazepam and then etizolam, clonazolam, flubromazolam, flualprazolam and flubromazepam.

**Table II:** Summary of Concentrations of All Designer Benzodiazepines Found in Living Cases (*N* = 554)

	Number of detections^a^	Concentrations (mg/L)					
		Median	Min.	Max.	25th percentile	75th percentile	Mono cases^b^
Clonazolam	22	0.0041	0.0017	0.053	0.0027	0.0090	0
Diclazepam	334	0.0096	0.0016	0.25	0.0045	0.019	16
Etizolam	40	0.054	0.015	0.30	0.025	0.10	3
Flualprazolam	10	0.0080	0.0033	0.056	0.0041	0.025	1
Flubromazepam	5	0.037	0.0070	0.70	0.017	0.44	1
Flubromazolam	20	0.0056	0.0004	0.036	0.0015	0.015	0
Phenazepam	138	0.022	0.0018	0.85	0.0082	0.087	4

^a^ Note that more than one designer benzodiazepine might be present in a case.

^b^ No other drug with relevance for impairment was detected.

In 25 of the 554 living cases, either diclazepam, etizolam, flualprazolam, flubromazepam or phenazepam were the only drugs detected, or the concentration of other detected drugs was considered not to be relevant for impairment. Most of these cases involved apprehended drivers. The concentrations of the respective benzodiazepines and the results of the CTI are presented in Table III. The physician concluded with mild, moderate or considerable impairment in 19 of the cases, whereas in 6 cases the conclusion of the CTI was that the subject was not impaired. In addition, the conclusion of the CTI was lacking, or it was impossible to conclude in four cases where a designer benzodiazepine was the only drug detected. These cases are not included in the table.

**Table III:** Living Cases Where Designer Benzodiazepines (DBZ) Were the Only Drugs Detected, or the Concentration of Other Detected Drugs Were Considered not to be Relevant to Impairment

#	Age (years)	Case type	Conclusion CTI	Type of DBZ	Conc. (mg/L)	Other drug detected (mg/L)
1	25–29	DUID	Moderately impaired	Diclazepam	0.061	Ethanol 0.053^a^
2	35–39	DUID	Considerably impaired	Diclazepam	0.048	
3	30–34	DUID	Moderately impaired	Diclazepam	0.045	Ethanol 0.084^a^
4	20–24	DUID	Mildly impaired	Diclazepam	0.035	Lorazepam^b^ 0.014
5	25–29	DUID	Considerably impaired	Diclazepam	0.035	THC 0.0011
6	20–24	DUID	Moderately impaired	Diclazepam	0.032	
7	30–34	DUID	Not impaired	Diclazepam	0.032	Lorazepam^b^ 0.012
8	<20	DUID	Moderately impaired	Diclazepam	0.019	
9	45–49	DUID	Moderately impaired	Diclazepam	0.016	Lorazepam^b^ 0.063
10	30–34	DUID	Considerably impaired	Diclazepam	0.014	
11	50–54	DUID	Moderately impaired	Diclazepam	0.011	Nitrazepam 0.017
12	20–24	DUID	Not impaired	Diclazepam	0.0089	
13	30–34	DUID	Not impaired	Diclazepam	0.0077	
14	20–24	DUID	Mildly impaired	Diclazepam	0.0077	THC 0.0007
15	35–39	DUID	Not impaired	Diclazepam	0.0054	
16	20–24	DUID	Mildly impaired	Diclazepam	0.0051	
17	25–29	DUID	Mildly impaired	Etizolam	0.21	
18	<20	DUID	Mildly impaired	Etizolam	0.12	Tramadol 0.071
19	40–44	DUID	Considerably impaired	Etizolam	0.11	
20	35–39	DUID	Considerably impaired	Flualprazolam	0.015	Tramadol 0.065
21	20–24	DUID	Not impaired	Flubromazepam	0.0070	Amphetamine 0.040
22	20–24	Other	Moderately impaired	Phenazepam	0.26	THC 0.0007
23	20–24	DUID	Moderately impaired	Phenazepam	0.17	
24	<20	DUID	Not impaired	Phenazepam	0.12	
25	40–44	DUID	Mildly impaired	Phenazepam	0.012	

^a^ Concentrations in g/L.

^b^ See Discussion for considerations regarding lorazepam as a metabolite of diclazepam.

**Figure 2:**
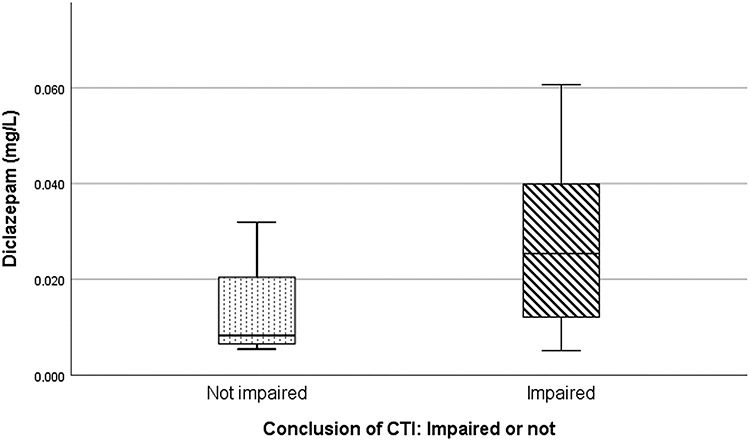
Concentration of diclazepam in relation to the conclusion of the CTI (‘impaired’ versus ‘not impaired’). The horizontal line in each box represents the median concentration (‘not impaired’ = 0.0083 mg/L, ‘impaired’ = 0.025 mg/L). The box lengths represent the 25–75 percentiles, whereas the whiskers represent the minimum and maximum values.

There are 16 mono cases concerning diclazepam in this data material. Impairment was seen in 12 of the 16 cases. Impairment was seen in most mono diclazepam cases (91%) with a concentration above 0.01 mg/L (median concentration of all living diclazepam cases). Figure 2 shows the concentration of diclazepam in relation to the conclusion of the CTI (‘not impaired’ versus mild, moderate or considerable impairment, grouped together as ‘impaired’). Although not statistically significant (*p* = 0.133), the concentration appeared higher in subjects where the physician concluded with impairment (mild, moderate or considerable). There was, however, a significant correlation between the concentration of diclazepam and the degree of impairment (Spearman’s rho = 0.561, *p* = 0.024). For the other drugs, too few mono cases were present to perform any statistical analyses.

**Figure 3:**
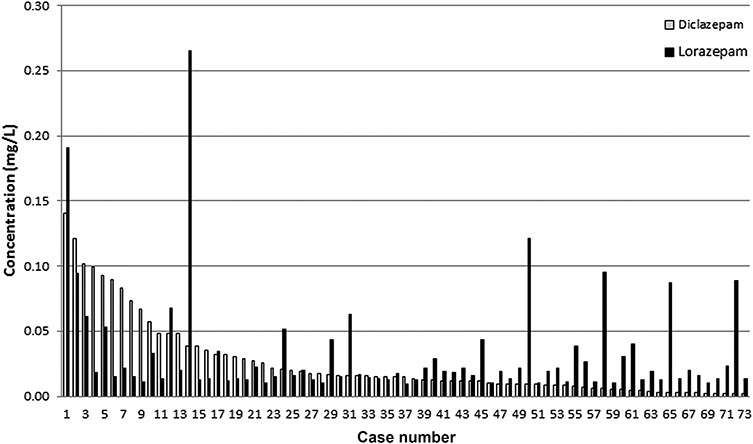
Concentrations for diclazepam and lorazepam for cases positive for both compounds (*N* = 73).

Lorazepam, which is both an active metabolite of diclazepam and a separate drug, was detected in 73 (22%) of the diclazepam positive living cases. The cut-off for lorazepam is 0.0096 mg/L and the cut-off for diclazepam is 0.0016 mg/L. Some of the lorazepam concentrations were quite considerable compared to the diclazepam concentration, as seen in Figure 3.

The benzodiazepines were also found in 21 autopsy cases. The age distribution of the subjects was slightly more disperse (all subjects were between 20 and 59 years), and there were almost as many women (48%) as men. The number of detections of each drug and the individual concentrations of each benzodiazepine in the autopsy cases are presented in Table IV. Other drugs were detected in all but one case.

Amphetamine, THC, clonazepam and methamphetamine were the most frequently detected other drugs in both types of cases, as shown in Table V.

## Discussion

This study documented blood concentrations of designer benzodiazepines in 575 cases, and the concentrations are related to impairment in a part of the material. The different designer benzodiazepines are discussed separately in the following sections. Postmortem blood concentrations are discussed briefly at the end. Blood to plasma concentration ratio is not known for the designer benzodiazepines in our study. For several traditional benzodiazepines, such ratios can be found in the literature, for example, the blood to plasma concentration ratio is 0.8 for alprazolam (34), 0.5–0.6 for clonazepam (35), 0.6 for diazepam (36) and 1.0–1.1 for nitrazepam (37). If the ratios are similar for the seven benzodiazepines in this study is, however, yet to determine.

### Living cases (apprehended drivers, other drug offences)

Diclazepam was the most frequent designer benzodiazepine detected in this study. Previous data regarding human blood concentrations of diclazepam are scarce. Rudolph et al. have reported a case of polydrug intoxication where diclazepam was found in a serum concentration of 0.057 mg/L (11). Several other designer benzodiazepines were also found in the case. After an intake of what was assumed to be 1 mg of diclazepam, a peak concentration of 0.0034 mg/L was seen in the blood of the single test subject (26). The study subject did not report any effects. This is comparable to the lower concentrations of diclazepam seen in this study and is just above the cut-off value in our method. In a previous study of Høiseth et al. of mostly apprehended drivers, a median concentration of 0.013 mg/L was seen in blood (22). The median concentration of 0.010 mg/L found in the present study is quite similar to this.

**Table IV:** Summary of Concentrations of All Designer Benzodiazepines in Autopsy Cases (*N* = 21)^a^

	Number of cases	Concentrations (mg/L)				
		Median	Min.	Max.	25th percentile	75th percentile
Diclazepam	13	0.0032	0.0018	0.032	0.0021	0.012
Etizolam	2	0.026	0.022	0.029	—	—
Flubromazolam	1	0.052	—	—	—	—
Phenazepam^b^	5	0.0059	0.0051	0.052	0.0054	0.036

^a^ Clonazolam, flualprazolam and flubromazepam were not detected in the autopsy cases.

^b^ Phenazepam was the only drug detected in one of these cases.

Diclazepam was the only drug detected or was found to be the main contributor to impairment, in 16 cases in this study. The results from the CTI in these cases indicate that diclazepam concentrations above 0.010 mg/L (median concentration of all living cases) are most often followed by impairment. A correlation between the concentration of diclazepam and the degree of impairment was found, as expected. This has been shown for other benzodiazepines earlier (38).

**Table V:** Number of Detections and Concentrations of the Other Drugs Most Frequently Detected in All the Designer Benzodiazepine Cases [Living cases (*N* = 554) and Autopsy Cases (*N* = 21)]

	DUID cases/other offences			Autopsy cases		
	Number of detections	Median conc. (mg/L)	IQR^a^	Number of detections	Median conc. (mg/L)	IQR^a^
Amphetamine	327	0.31	0.55	9	0.39	1.2
THC (tetrahydrocannabinol)	275	0.0027	0.0042	11	0.0056	0.0093
Clonazepam	201	0.015	0.028	5	0.026	0.028
Methamphetamine	117	0.23	0.49	5	0.44	1.6
N-desmethyldiazepam^b^	111	0.16	0.24	5	0.094	0.71
Alprazolam	89	0.020	0.033	4	0.023	0.065
Ethanol^c^	88	1.0	1.4	5	0.74	0.89
Diazepam	86	0.22	0.29	4	0.13	2.6
Lorazepam^d^	75	0.018	0.016	—	—	—
MDMA (ecstasy)	56	0.20	0.26	2	0.44	—
Cocaine	35	0.066	0.10	2	0.019	—

^a^ Interquartile range.

^b^ Metabolite of diazepam.

^c^ Concentrations in g/L.

^d^ Metabolite of diclazepam.

Lorazepam is a metabolite of diclazepam but is also a separate drug commonly prescribed to treat psychiatric illnesses. There are little data regarding which concentrations of lorazepam to expect after an intake of diclazepam. In the study of diclazepam by Moosmann et al., the main metabolites observed in blood were delorazepam, lormetazepam and lorazepam (26). A single case study by Lehmann et al. found postmortem concentrations of diclazepam and its metabolites in different tissues and body fluids (39). Based on the concentrations seen in these studies, it is expected that the concentration of lorazepam can be significant in blood in the terminal phase after an intake of diclazepam (26). Therefore, previous studies might support some of the high lorazepam-to-diclazepam concentration ratios seen in this study as a result of diclazepam intakes, although a separate intake of lorazepam is suspected in some cases with exceptionally high ratios.

Phenazepam has been used clinically, mostly in Russia, and adult doses are normally 0.5 mg 2–3 times daily for the treatment of anxiety (40). After oral dosing with 3 or 5 mg of phenazepam, peak plasma concentrations were 0.024 and 0.038 mg/L, respectively (41). We have not found any articles describing the plasma or serum to blood concentration ratio for phenazepam, so it is not possible to directly compare concentrations in plasma, serum and blood. Kerrigan et al. have reported a case of an apprehended driver with profound psychomotor impairment and slurred speech. Only phenazepam was detected in blood at a concentration of 0.076 mg/L (24). Dargan et al. have published a case where a man was hospitalized with prolonged confusion and disorientation. The serum concentration of phenazepam was 0.49 mg/L, and no other drug was detected (42). There are also published data from apprehended drivers in Georgia, USA. The blood concentrations of phenazepam ranged from 0.04 to 3.2 mg/L, with a median of 0.17 mg/L (23). In five of those cases phenazepam was the only drug detected, and there were observations indicating impairment. Kriikku et al. have published findings from apprehended drivers (*n* = 141) in Finland (43). The median phenazepam concentration in blood was 0.061 mg/L (range 0.004–3.6 mg/L). The phenazepam concentrations in the present study (median 0.022 mg/L and maximum concentration 0.85 mg/L) are therefore somewhat lower than what is previously published in DUID cases. There were four mono cases with phenazepam in our study, with concentrations ranging from 0.012 to 0.26 mg/L. Impairment was seen in three of the cases.

Etizolam have been used clinically as a sedative–hypnotic drug in certain European and Asian countries since the 1980s, and adult doses may range from 0.5 to 3 mg per day (44). Concentrations of 0.0083 and 0.018 mg/L are reported after single intake of 0.5 or 1 mg etizolam, respectively, in healthy volunteers (45–47). After 0.5 mg etizolam twice daily for a week, the peak plasma concentration after the last dose was 0.0093 mg/L (45). In an experimental study (*n* = 16), a single administration of etizolam doses of up to 1 mg had no significant effect on vigilance, short-term memory, psychomotor coordination or speed in decision-making (48). No pharmacokinetic measurements were made. The median concentration of etizolam found in our study of 0.054 mg/L is similar to the concentrations found in a previous study (22), but the maximum concentration in our study is somewhat higher. There are little data regarding toxic concentrations after intake of etizolam, but somnolence was reported in a case where etizolam (0.28 mg/L) was identified in serum in addition to clonazolam (0.010 mg/L) (49). The plasma to blood concentration ratio is not known for etizolam. There were three mono cases with etizolam in this study, with concentrations ranging from 0.11 to 0.21 mg/L in blood. The physician concluded with impairment in all three cases. ‘Considerable impairment’ was seen in the case with the lowest blood concentration, and ‘mild impairment’ was the conclusion in the two other cases with higher concentrations, which probably reflect differences in tolerance to the drug. Tolerance to the effects of benzodiazepines is expected, as studies suggest (partial) tolerance to cognitive effects after long-term use (50).

Users of flubromazolam have described the drug as very potent and with sedating effects that may last for several days (51). An intake of 0.5 mg flubromazolam gave strong sedation, and a maximum serum concentration of 0.0086 mg/L was seen (25). There is a case report where flubromazolam was ingested 48 hours prior to hospitalization (2 mg) and 19 hours prior to hospitalization (3 mg). The serum concentration 19 hours after the last ingestion of flubromazolam was 0.059 mg/L, and the patient was then in a deep coma (14). Although not directly comparable, the maximum concentration seen in our data material was 0.036 mg/L in blood. The median blood concentration was 0.0056 mg/L in this study as compared to the median blood concentration of 0.012 mg/L seen in the previous study of Høiseth et al. (22).

Oral consumption of 4 mg flubromazepam in one volunteer gave a peak concentration of 0.078 mg/L in serum (27). The subject experienced some fatigue and enhanced need of sleep for 3 days after the intake, but no other effects were observed. This concentration is somewhat higher than the median blood concentration in both this study (0.037 mg/L) and a previous study (0.055 mg/L) (22), but it should be noted that the plasma to blood concentration ratio is not taken account for in this comparison.

We found no experimental studies regarding pharmacokinetic data after ingestion of clonazolam. In a near-fatal poly intoxication, clonazolam was found in serum at 0.0068 mg/L (13). A clonazolam concentration of 0.010 mg/L in serum, combined with a relatively high etizolam concentration, was also found in a somnolent man admitted to hospital (49). Høiseth et al. reported a median blood clonazolam concentration of 0.0053 mg/L in a previous study of mostly DUID cases (22), which is similar to the median concentration of clonazolam found in this study (0.0041 mg/L).

Flualprazolam is an emerging drug on the illicit drug market. Papsun et al. recently published data from apprehended drivers in the USA (9). In Sacramento, California, the blood concentrations found ranged from 0.005 to 0.15 mg/L (*n* = 123), with a median of 0.018 mg/L. The other reported blood concentrations were from multiple states and ranged from 0.0083 to 0.068 mg/L (*n* = 20), with a median of 0.012 mg/L. In comparison, the median blood concentration seen in our study was 0.0080 mg/L. There was one mono case in our study involving flualprazolam. The concentration of flualprazolam in this case was 0.015 mg/L, and the conclusion of the CTI was ‘considerably impaired’.

### Autopsy cases

The designer benzodiazepines diclazepam, etizolam, flubromazolam and phenazepam were found in 21 autopsy cases in this study. Other drugs were detected in all cases but one, but the cause of death is not known to the authors in any of these cases due to data protection regulations. The contribution of the designer benzodiazepines is therefore not known. Except for flubromazolam, the concentrations detected are relatively similar to what is seen in living cases.

There exist some reports of diclazepam found in postmortem blood in addition to other psychoactive drugs, with diclazepam concentrations ranging from <0.001 to 0.070 mg/L (15, 19, 39). Diclazepam was found in 13 autopsy cases in the present study, with blood concentrations ranging from 0.0018 to 0.032 mg/L.

More information is available for postmortem concentrations of phenazepam. In a Scottish material concerning medico-legal autopsies, there were two cases where phenazepam was noted as the cause of death. The concentrations in femoral blood were 1.2 and 1.6 mg/L. There were 54 cases where phenazepam was detected in combination with other drugs, and the cause of death was thought to be drug related. In these cases phenazepam ranged from <0.005 to 0.9 mg/L (median 0.10 mg/L) (20). Kriikku et al. have reported a median blood concentration of 0.048 mg/L (range 0.007–1.6 mg/L) in 17 medico-legal autopsies in Finland. In none of the cases, phenazepam was considered to be the sole cause of death (43). Phenazepam was also found in postmortem blood in 29 cases reported by Crichton et al. In most cases, phenazepam was not directly related to the cause of death, but in two cases the drug was either contributing or the certified cause of death (blood concentration 0.97 and 1.64 mg/L) (52). Others have also reported cases where phenazepam was found, in combination with other drugs, at concentrations ranging from 0.22 to 2.52 mg/L (53–55). In comparison, the concentration of phenazepam found in the five autopsy cases in our study ranged between 0.0051 and 0.052 mg/L, which is considerably lower than the concentrations reported in the few cases where the drug was considered the sole cause of death.

Etizolam have been identified in the blood of several fatal intoxications in combinations with other drugs. Reported concentrations range from 0.01 to 0.27 mg/L (18, 19, 54, 56, 57). There is also a case where the deceased had drowned and no other drug was detected. Etizolam was found in a concentration of 0.26 mg/L (12). There were two detections of etizolam in postmortem blood in our study, ranging from 0.022 to 0.029 mg/L. Other drugs were present as well in the blood samples.

In this study, there was one case where flubromazolam was detected in postmortem blood in a concentration of 0.052 mg/L. Additional drugs were also detected. There are some reports of flubromazolam in combination with other drugs in postmortem blood. Mei et al. reported a concentration of flubromazolam of 0.040 mg/L in heart blood (19). In two reports of methadone-associated deaths, flubromazolam was also reported in femoral blood at 0.0044 and 0.0080 mg/kg (58). The concentration detected in this study (0.052 mg/L) is similar to the serum concentration of flubromazolam seen in a case where the sample giver was in a deep coma (14). However, little is known about postmortem changes of flubromazolam.

### Strengths and limitations

The present study adds information about concentrations in both living subjects and postmortem cases. The strength of the present study was the large size of the material. A weakness of the present study is, however, that there are still a limited number of mono cases, thus a small material for comparing blood concentrations and impairment. There is also no information regarding time of consumption, previous history of drug use or dosing, but this is difficult to obtain in this kind of study. The CTI was done by several different physicians throughout the country, and the authors have no information regarding their experience with evaluating impairment and thereby the validity of the conclusion of the CTI. The cause of death is not known in the autopsy cases, which makes it hard to determine the role of the designer benzodiazepines in these cases.

## Conclusion

This study consists of a large data material concerning seven designer benzodiazepines, in particular diclazepam, phenazepam and etizolam. Detection of these drugs represents mostly (if not strictly) illicit use in Norway. There are several mono cases where concentrations of the designer benzodiazepines can be related to impairment. Generally, there are few published studies reporting concentrations of designer benzodiazepines. The presented blood concentrations can be helpful with interpretation of cases involving one or more of the seven designer benzodiazepines included in this study. The results indicate that concentrations commonly observed in forensic cases are associated with impairment.

## Supplementary Material

bkaa043_Supp
