# Supplementary material for: Blood Concentrations of Designer Benzodiazepines: Relation to Impairment and Findings in Forensic Cases
Source: J Anal Toxicol. 2020 May 5;44(8):905–14. doi: 10.1093/jat/bkaa043 (PMC7733327; doi:10.1093/jat/bkaa043)
Supplement: bkaa043_Supp [file bkaa043_supp.zip › jat-19-3044-File006_bkaa043.doc]

**Supplementary Table I.** Per se limits in Norway for driving under the influence of drugs.

*Converted from µmol/L

| **Drug** | **Per se limit (mg/L)*** |
| --- | --- |
| *Benzodiazepines and similar drugs* |  |
| Alprazolam | 0.032 |
| Bromazepam | 0.316 |
| N-desmethyldiazepam | 1.478 |
| Diazepam | 0.702 |
| Etizolam | 0.117 |
| Phenazepam | 0.014 |
| Flunitrazepam | 0.016 |
| Clobazam | 1.995 |
| Clonazepam | 0.013 |
| Lorazepam | 0.093 |
| Nitrazepam | 0.213 |
| Oxazepam | 2.093 |
| Triazolam | 0.003 |
| Zolpidem | 0.325 |
| Zopiclone | 0.077 |
| *Cannabis* |  |
| Tetrahydrocannabinol (THC) | 0.013 |
| *GHB* |  |
| Gamma-hydroxybutyric acid (GHB) | 961 |
| *Hallucinogens* |  |
| Ketamine | 0.841 |
| Lysergic acid diethylamide (LSD) | 0.009 |
| *Opioids* |  |
| Buprenorphine | 0.002 |
| Methadone | 0.259 |
| Morphine | 0.105 |
| Oxycodone | 0.159 |
| *Stimulants* |  |
| Amphetamine | 2.219 |
| Cocaine | 0.264 |
| 3,4-methylenedioxymethamphetamine (MDMA) | 2.587 |
| Methamphentamine | 2.010 |
| Methylphenidate | 0.064 |
